# Promoting Aspirin Use for Cardiovascular Disease Prevention Among an Adult Internet-Using Population: A Pilot Study

**DOI:** 10.3389/fpubh.2021.500296

**Published:** 2021-03-16

**Authors:** Niki C. Oldenburg, Keith J. Horvath, Jeremy Van't Hof, Jeffrey R. Misialek, Alan T. Hirsch

**Affiliations:** ^1^Lillehei Heart Institute and Cardiovascular Division, University of Minnesota Medical School, Minneapolis, MN, United States; ^2^Division of Epidemiology and Community Health, School of Public Health, University of Minnesota, Minneapolis, MN, United States

**Keywords:** health education, internet, aspirin, cardiovascular disease, prevention

## Abstract

Cardiovascular disease prevention strategies include aspirin use as a preventive measure. The internet can be used to raise public awareness, promote healthy lifestyles, and improve disease management. This pilot study describes the feasibility of an educational website to recruit and follow adult internet users to examine whether they talked to their physician about aspirin and initiated aspirin use. As part of a statewide intervention promoting an aspirin regimen to prevent heart attacks and strokes in Minnesota, visitors to the website were encouraged to complete an aspirin candidacy tool. Between October, 2015 and February, 2016, men 45–79 and women 55–79 who identified as aspirin candidates were invited to participate in a 6-month study involving four, 5 min online surveys to examine physician discussions about aspirin, aspirin use, and mobile technology use. During the 5-month recruitment period, 234 adults enrolled in the study. Of the 174 who completed the baseline survey and at least one follow-up survey, 74 (43.5%) did not use aspirin at baseline. During follow-up, 12 (16.2%) talked to their doctor about aspirin and 31 (41.8%) initiated aspirin use. Internet, social media, and mobile technology use were high among this population. An educational website may have provided a cue to action for aspirin discussions with physicians and aspirin initiation. More research is needed to evaluate the utility of on-line tools to increase appropriate aspirin use among internet-using populations.

## Introduction

Cardiovascular disease (CVD) prevention strategies have utilized the internet to raise public awareness, promote healthy lifestyles, and improve disease management among the population of internet users that includes over 90% percent of U.S. adults (1). Much of the research surrounding internet-based CVD interventions has focused on individually tailored, interactive interventions among participants recruited primarily from work sites or medical clinics. The health outcomes measured have spanned risk reduction goals (smoking cessation, increased physical activity, and dietary control) to disease management goals (medical appointment adherence, medication compliance, and self-care strategies) (2–7). Little is known about the impact internet-based CVD interventions may have on the behaviors of community-based internet users. In addition, it is unclear whether internet users who are middle-aged or older are willing to participate in online research.

As part of a large public health campaign promoting aspirin use to reduce heart attacks and strokes, an educational website and aspirin candidacy tool were created to prompt aspirin discussions with physicians and aspirin initiation when indicated. The aims of this study are to assess among those individuals identified as potential aspirin candidates from the online self-assessment the: (1) feasibility of recruiting and retaining a cohort of middle-aged to older internet users; (2) computer and mobile technology use of these individuals; and (3) proportion of these individuals who seek advice from their physician and who adopt a preventive aspirin regime.

## Method

### Community-Based Intervention

**Figure 1:**
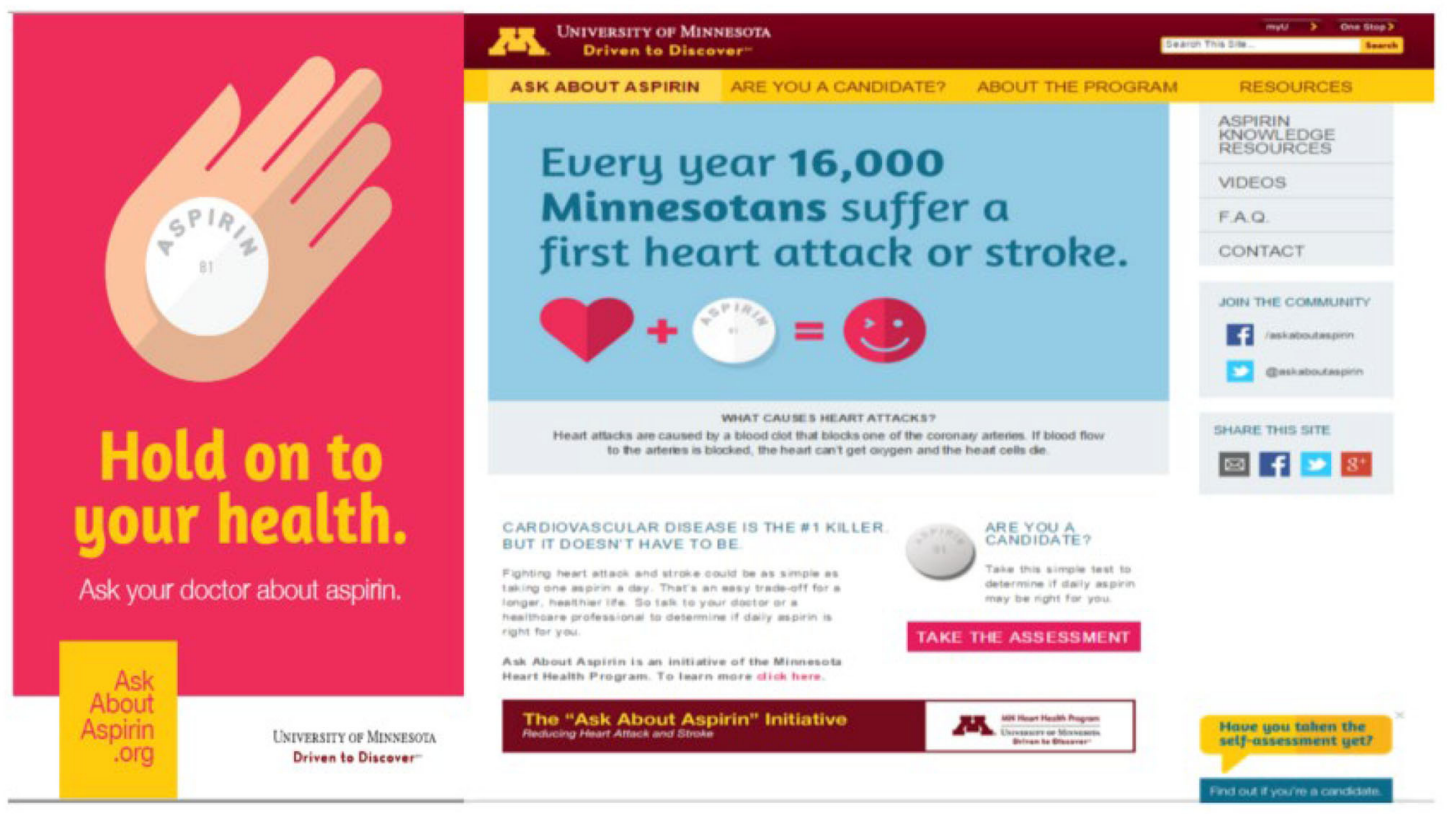
Example of “Ask About Aspirin” advertisement and website.

A multi-year, state-wide, media and health professional campaign was launched in 2012 in Minnesota to promote the 2009 U.S. Preventive Services Task Force (USPSTF) recommendations on aspirin use for the primary prevention of cardiovascular disease (8). The media campaign included radio spots, billboards, print and online advertisements (Facebook, Twitter, Pandora, Google Adwords), and brochures that encouraged individuals to talk to their doctor about a preventive aspirin regimen. Simultaneously, the media campaign directed individuals to visit the “Ask About Aspirin” educational website for more information about aspirin and to find out if they are an aspirin candidate (Figure 1). Adults in the 2009 USPSTF candidacy range (men 45–79 years; women 55–79 years) were encouraged to complete an aspirin candidacy tool that consisted of 9 questions addressing age, sex, CVD history, and contraindications to aspirin use. Those identified as potential aspirin candidates learned that aspirin may be beneficial for them and were advised to speak with their physicians about initiating a preventive aspirin regime.

### Study Design

**Figure 2:**
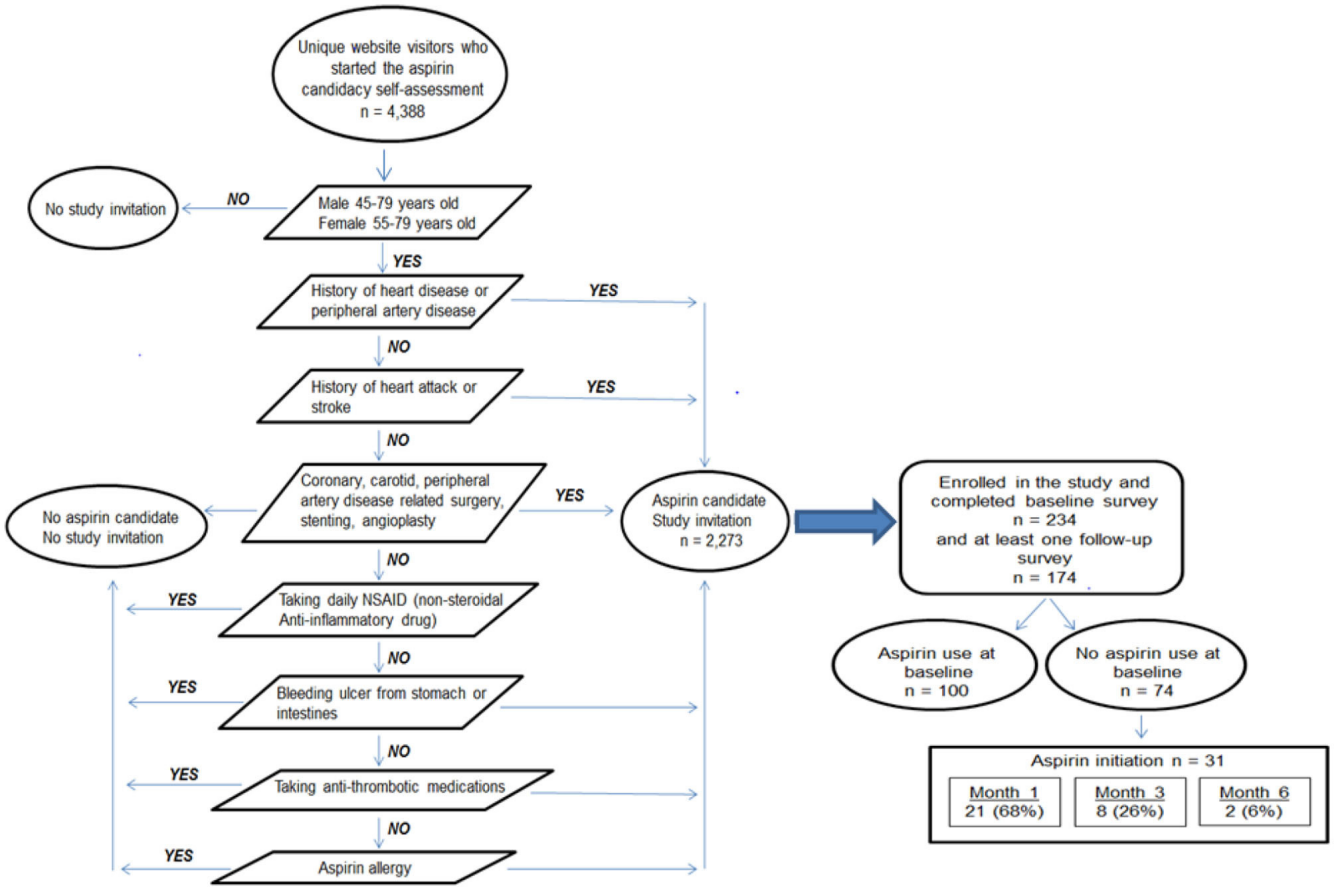
Aspirin candidacy, study invitation, and aspirin initiation among visitors to the educational website.

**Figure 3:**
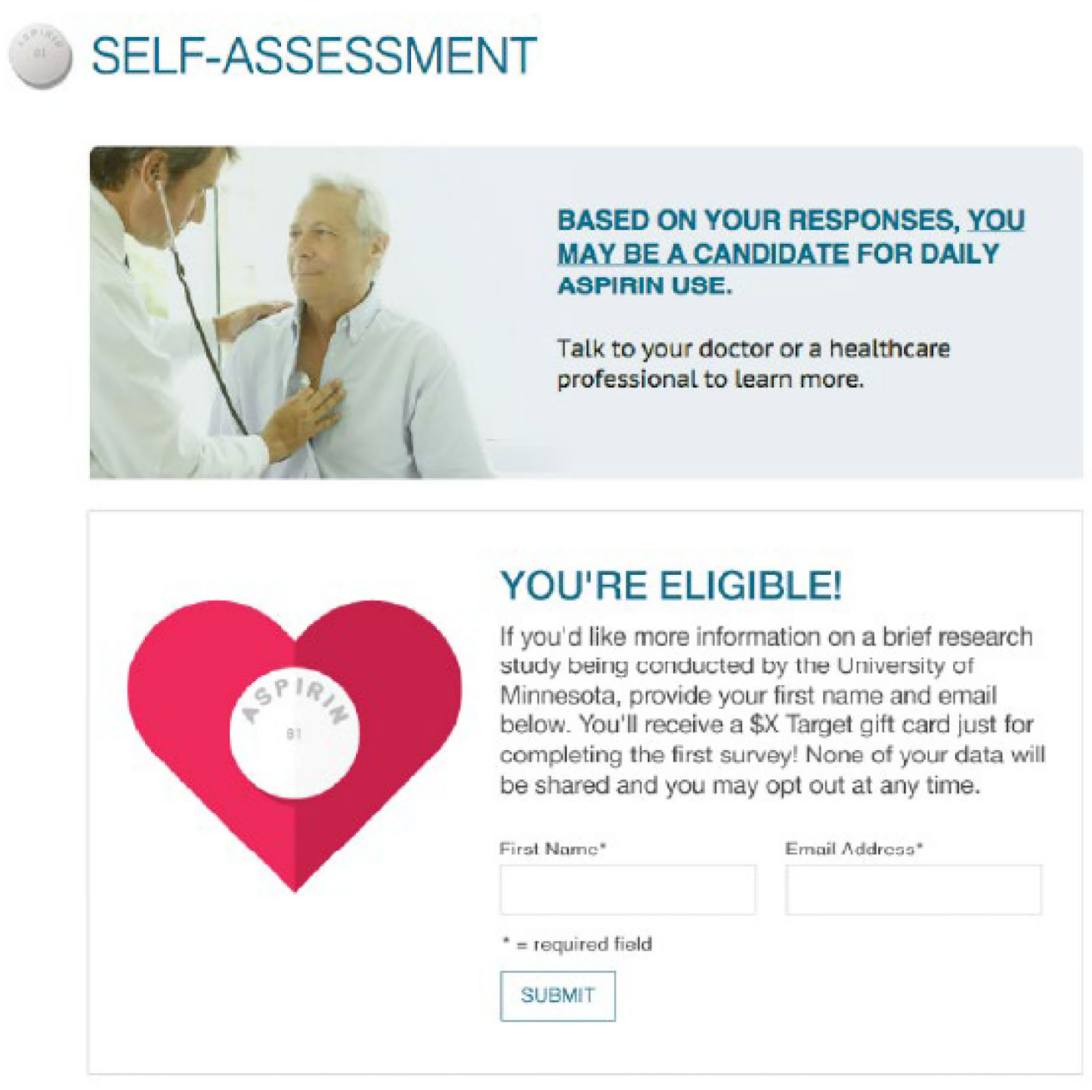
Example of study invitation upon completing the aspirin candidacy tool. This image was updated when the study invitation went “live” on the website to include the exact dollar amounts and the type of gift card (amazon.com rather than Target gift cards were used).

Between October, 2015 and February, 2016, adults who completed the aspirin candidacy tool, identified as aspirin candidates, and who were men 45–79 years and women 55–79 years old (Figure 2) were invited to participate in a 6-month online study about their aspirin-related behaviors and mobile technology use via a pop-up box on the website (Figure 3). To learn more about the study, interested individuals provided their first name and email address to facilitate a point of contact. Confidentiality was assured at the point of contact and was expanded upon in the on-line consent form. Once informed consent about the study was obtained from participants, they were asked to complete four, 5 min online surveys across a 6-month follow-up period (baseline and 1-, 3-, and 6-months).

Survey questions addressed sex (male/female), age (age reported in years at time of survey), aspirin discussions with physicians, aspirin use, and mobile technology and social media use. Aspirin discussions were determined by a “Yes/No” response to the baseline question “Have you ever talked with your doctor or other health professional about whether you should use aspirin as a means for preventing a heart attack or stroke?” In the follow-up surveys, this question began with the phrase “Since the last online survey you completed x month(s) ago…” to assess temporality. Aspirin use was determined by a “Yes/No” response to the question “Do you currently take aspirin to prevent a heart attack or stroke?”

Participants received a $5 Amazon.com gift card via email for each survey they completed during the first 3-months, and a $10 Amazon.com gift card for the final survey at month 6. Links to the online surveys were emailed at each time point, followed by 2 reminder emails within a week if surveys had not been completed. Qualtrics (Qualtrics, Provo, UT) was used as the online survey management software. Data analyses included logistic regression (sex and baseline aspirin use), a two sample *t*-test (age and baseline aspirin use), and Pearson's chi-square tests (media use and baseline aspirin use) and were conducted using Stata version 12 (StataCorp LP, College Station, Texas). The University of Minnesota's Institutional Review Board, responsible for the ethical conduct of human research, approved the study.

## Results

During the 5-month enrollment period, there were 32,584 unique visits to the “Ask About Aspirin” website of which 4,388 visitors started the aspirin candidacy tool. Two thousand two hundred and seventy three completed the assessment and were identified as aspirin candidates which prompted a study invitation; 234 (10.2%) enrolled in the study and completed a baseline survey. Of these 234 participants, 149 (63.6%) completed the survey at month 1, 140 (59.8%) at month 3, and 123 (52.5%) at month 6. More women than men participated (58.0 and 41.9%, respectively) and the mean age was 61.9 years (range, 46–79 years).

When restricting analyses to the 233 participants who answered “Yes” or “No” to the aspirin use question, 59 (25.3%) completed only the baseline survey, 29 (12.4%) completed 2 surveys, 52 (22.3%) completed 3 surveys, and 93 (39.9%) completed all 4 surveys. At baseline, men were more likely to be aspirin users than women (OR = 2.73, 95% CI = 1.44, 5.18); age was not significantly different between aspirin users and non-users, 62.5 and 61.0 mean ages, respectively (*t* = −1.4476, *p* = 0.15).

One hundred and seventy four participants completed both the baseline survey and at least one follow-up survey, with 100 (57.4%) reporting current aspirin use on the baseline survey. Of the 74 participants who did not use aspirin at baseline, 31 (41.8%) initiated aspirin use to prevent a heart attack or stroke and 12 (16.2%) talked to their doctor about aspirin use during the follow-up period. The majority of these 31 individuals started aspirin in the first month (Figure 2). When examining the 31 aspirin initiators further, 12 (38.7%) talked to their doctor about aspirin use during follow-up, 9 (29.0%) had discussed aspirin use with their doctor prior to baseline, 6 (19.3%) initiated aspirin use on their own, and 4 (12.9%) took aspirin previously, stopped, and then restarted during the study. Of the 100 participants who reported aspirin use at baseline, 96 (96.0%) continued using aspirin during follow-up.

The majority of participants accessed the internet and used social media sites several times a day. A majority also used laptops or tablets and used cell phones to access the internet. Eighty-three percent used cell phones to access the internet and 87% used social media. No difference in technology use existed between the baseline aspirin users and aspirin non-users (Table 1).

**Table 1:** Computer or mobile technology use among participants by baseline aspirin use.

**Survey questions regarding computer and mobile technology use**	**Total cohort (*n* = 174)**	**ASA non-users (*n* = 74) *n* (%)**	**ASA users (*n* = 100) *n* (%)**	**Chi-square**
**Which of the following devices do you use at least once a month?**				
Desktop computer	107 (61.4%)	46 (62.1%)	61 (61.0%)	0.88
Laptop computer or notebook	116 (66.6%)	49 (66.2%)	67 (67.0%)	0.91
Tablet computer	117 (67.2%)	48 (64.8%)	69 (69.0%)	0.57
How often did you use Internet or email?^*^				
Several times a day	150 (86.2%)	62 (83.7%)	88 (88.0%)	0.44
About once a day or less	22 (12.6%)	11 (14.8%)	11 (11.0%)	
**Which of the following types of social media have you ever used?**				
Facebook	144 (82.7%)	61 (82.4%)	83 (83.0%)	0.92
LinkedIn	79 (45.4%)	36 (48.6%)	43 (43.0%)	0.46
Pinterest	76 (43.6%)	38 (51.3%)	38 (38.0%)	0.08
Instagram	29 (16.6%)	10 (13.5%)	19 (19.0%)	0.34
Twitter	37 (21.2%)	17 (22.9%)	20 (20.0%)	0.64
**In the past month, about how often did you use any kind of social media, like Facebook, LinkedIn, Pinterest, Instagram, or Twitter?**				
Several times a day	80 (45.9%)	33 (44.5%)	47 (47.0%)	0.57
About once a day	42 (24.1%)	17 (22.9%)	25 (25.0%)	
3–5 days a week or less	29 (16.6%)	15 (20.2%)	14 (14.0%)	
Have you ever used your cell phone to send or receive email?^*^				
Yes	136 (78.1%)	63 (85.1%)	73 (73.0%)	0.08
No	34 (19.5%)	10 (13.5%)	24 (24.0%)	
Have you ever used cell phone to send or receive text messages?^*^				
Yes	160 (91.9%)	70 (94.5%)	90 (90.0%)	0.39
No	10 (5.7%)	3 (4.0%)	7 (7.0%)	
On average, how many text messages do you send and receive in a month?^*^				
1–20	36 (20.6%)	14 (18.9%)	22 (22.0%)	0.62
21–50	41 (23.5%)	17 (22.9%)	24 (24.0%)	
51–100	39 (22.4%)	16 (21.6%)	23 (23.0%)	
>100	40 (22.9%)	21 (28.3%)	19 (19.0%)	
Have you ever used your cell phone to access the internet?^*^				
Yes	144 (82.7%)	65 (87.8%)	79 (79.0%)	0.22
No	25 (14.3%)	8 (10.8%)	17 (17.0%)	

* Percentages do not add up to 100% due to individuals who responded “Don't Know” or “Refused.”

## Discussion

The study showed that it is feasible to recruit and retain a middle-aged to older, internet-using population in an online study. Participation rates for individuals recruited via the internet have ranged between <1% to approximately 15% among teenagers, and young to middle-aged adults (9–14). Study retention rates among internet-based interventions ranged widely, with follow-up rates of 13% to >80% (9, 10, 12, 15, 16). This study's participation rate of 10% and retention rate of 53% suggest that age may not be a barrier, as age-related differences in internet use are decreasing (17).

As may be expected among internet users, a higher percentage of the participants used smart phones and social media than the general adult population. Among U.S. adults, 79% of individuals ages 50–64 and 53% of those 65 years and older own a smartphone compared to 83% in the current study sample. In addition, 69% of 50–64 year olds and 40% of individuals 65 years and older use social media compared to 87% in this study (18, 19). Individuals recruited from the internet may be more frequent users of other mobile technology and social media platforms, and thus may be more responsive to health interventions that leverage varied mobile and social media tools or outlets.

A majority of the participants were already using aspirin to prevent a heart attack or stroke at the start of the study. Given the website's focus on the benefits of preventive aspirin use, such individuals may have been seeking online health information to confirm their current health behaviors. Among participants who did not use aspirin at baseline, aspirin initiation increased most dramatically during the first month of follow-up. This uptake in aspirin use is noteworthy given the minimal interaction the website provided its visitors and the short time span in which aspirin initiation was achieved. Individuals seeking online health information may be motivated to improve their health, and thus more responsive to behavior change and health maintenance messaging.

Approximately two thirds of the participants, who initiated aspirin use during follow-up, had had an aspirin discussion with their physician either prior to or during the study. This may suggest that the website prompted them to either act upon previous aspirin discussions with their physicians or to initiate new ones. Positive associations between aspirin discussions with a physician and aspirin initiation have been shown in other studies (8, 20).

An unique opportunity may exist for public health campaigns to promote community-wide behavior change among internet-users and to monitor its impact. With internet use among older adults becoming nearly universal (1, 17), internet-based campaigns focusing on CVD prevention are likely to see increases in reach and impact among this population.

### Limitations

As a pilot study, the findings presented are primarily exploratory and hypothesis-generating. The lack of a control group and small numbers may limit the generalizability of the study findings to other middle-age to older internet users. Self-selection bias may be present since study participants may be more motivated to act upon internet-based health information than non-study participants. Aspirin initiation may be subject to self-report bias; however, this bias is likely minimal (21). It is not known whether aspirin initiators included individuals who should not be taking aspirin. The educational website and aspirin candidacy tool encouraged all individuals to ask their physician or health professional if a preventive aspirin regime was right for them.

## Conclusion

This study provides preliminary evidence that it is feasible to recruit and maintain a middle- to older aged population of internet users in an online study. In addition, this study suggests that an educational website and aspirin candidacy tool may provide a promising CVD prevention strategy to increase aspirin physician discussions and aspirin use. The frequent and widespread use of mobile technology and social media platforms in this study suggest that older adult internet users may also be amenable to more varied digital interventions. More rigorous studies, such as randomized controlled trials, are needed to determine the utility of this approach to increasing appropriate aspirin use among older U.S. adults.

## Data Availability Statement

The datasets generated for this study are available on request to the corresponding author.

## Ethics Statement

The studies involving human participants were reviewed and approved by University of Minnesota (# 1201M08921). Written informed consent for participation was not required for this study in accordance with the national legislation and the institutional requirements.

## Author Contributions

NO, KH, JV, and AH contributed to the study design, implementation, analysis, and manuscript writing. JM contributed to the statistical design and analysis and manuscript writing. All authors contributed to the article and approved the submitted version.

## Conflict of Interest

NO has received funding from a grant provided by the Council on Aspirin for Health and Prevention for a separate research study. The remaining authors declare that the research was conducted in the absence of any commercial or financial relationships that could be construed as a potential conflict of interest. The reviewer HB declared a shared affiliation, with the authors to the handling editor at the time of the review.

## References

[1] Pew Research Center. Internet/Broadband Fact Sheet. Available online at: http://www.pewinternet.org/fact-sheet/internet-broadband/ (accessed September 5, 2019).

[2] Devi R Singh SJ Powell J Fulton EA Igbinedion E Rees K. Internet-based interventions for the secondary prevention of coronary heart disease. Cochrane Database Syst Rev. (2015) 12:CD009386. 10.1002/14651858.CD009386.pub2 26691216 PMC10819100

[3] Free C Phillips G Galli L Watson L Felix L Edwards P. The effectiveness of mobile-health technology-based health behavior change or disease management interventions for health care consumers: a systematic review. PLOS Med. (2013) 10:e1001362. 10.1371/journal.pmed.1001362 23349621 PMC3548655

[4] Mathieu E McGeechan K Barratt A Herbert R. Internet-based randomized controlled trials: a systematic review. J Am Med Inform Assoc. (2013) 20:568–76. 10.1136/amiajnl-2012-001175 23065196 PMC3628055

[5] Cugelman B Thelwall M Dawes P. Online interventions for social marketing health behavior change campaigns: a meta-analysis of psychological architectures and adherence factors. J Med Inter Res. (2011) 13:e17. 10.2196/jmir.1367 21320854 PMC3221338

[6] Graham AL Carpenter KM Cha S Cole S Jacobs MA Raskob M. Systematic review and meta-analysis of Internet interventions for smoking cessation among adults. Subst Abuse Rehabil. (2016) 7:55–69. 10.2147/SAR.S101660 27274333 PMC4876804

[7] Kuijpers W Groen WG Aaronson NK van Harten WH. A systematic review of web-based interventions for patient empowerment and physical activity in chronic diseases: relevance for cancer survivors. J Med Inter Res. (2013) 15:e37. 10.2196/jmir.2281 23425685 PMC3636300

[8] Oldenburg NC Duval S Luepker RV Finnegan JR LaMarre H Peterson KA. A 16-month community-based intervention to increase asprin use for primary prevention of cardiovascular disease. Prev Chronic Dis. (2014) 11:130378. 10.5888/pcd11.130378 PMC4023687 24831287

[9] Cobb NK Poirier J. Effectiveness of a multimodal online well-being interventi on – a randomized controlled trial. Am J Prev Med. (2014) 46:41–8. 10.1016/j.amepre.2013.08.018 24355670

[10] Shorter GW Ferry F. Recruitment and retention in internet based randomised trials. Trials. (2013) 14:O113. 10.1186/1745-6215-14-S1-O113

[11] Bold KW Hanrahan TH O'Malley SS Fucito LM. Exploring the utility of web-based social media advertising to recruit adult heavy-drinking smokers for treatment. J Med Internet Res. (2016) 18:e107. 10.2196/jmir.5360 27194456 PMC4889869

[12] Villanti AC Jacobs MA Zawistowski G Brookover J Stanton CA Graham AL. Impact of baseline assessment modality on enrollment and retention in a Facebook smoking cessation study. J Med Internet Res. (2015) 17:e179. 10.2196/jmir.4341 26183789 PMC4527002

[13] Koo M Skinner H. Challenges of Internet recruitment: a case study with disappointing results. J Med Internet Res. (2005) 7:e6. 10.2196/jmir.7.1.e6 15829478 PMC1550633

[14] Isaacson RS Seifan A Haddox CL Mureb M Rahman A Scheyer O. Using social media to disseminate education about Alzheimer's prevention and treatment: a pilot study on Alzheimer's Universe (www.AlzU.org). J Comm in Healthcare. (2018) 11:106–13. 10.1080/17538068.2018.1467068 30740140 PMC6364853

[15] Foster C Grimmett C May CM Ewings S Myall M Hulme C. A web-based intervention (RESTORE) to support self-management of cancer-related fatigue following primary cancer treatment: a multi-centre proof of concept randomized controlled trial. Support Care Cancer. (2016) 24:2445–53. 10.1007/s00520-015-3044-7 26643072 PMC4846690

[16] Murray E White IR Varagunam M Godfrey C Khadjesari Z McCambridge J. Attrition revisited: adherence and retention in a web-based alcohol trial. J Med Internet Res. (2013) 15:e162. 10.2196/jmir.2336 23996958 PMC3815435

[17] U.S. Census Bureau. Computer and Internet Use in the United States: 2016. American Community Survey Reports. Available online at: https://www.census.gov/content/dam/Census/library/publications/2018/acs/ACS-39.pdf (accessed May 31, 2019).

[18] Pew Research Center. Mobile Fact Sheet. Available online at: http://www.pewinternet.org/fact-sheet/mobile (accessed September 5, 2019).

[19] Pew Research Center. Social Media Fact Sheet. Available online at: http://www.pewinternet.org/fact-sheet/social-media (accessed September 5, 2019).

[20] Van't Hof JR Duval S Misialek JR Oldenburg NC Jones C Eder M. Primary prevention aspirin use in an African American population: the impact of health beliefs and social norms. J Comm Health. (2019) 44:561–8. 10.1007/s10900-019-00646-5 PMC6504594 30895416

[21] Zantek ND Luepker RV Duval S Miller K Oldenburg N Hirsch AT. Confirmation of reported aspirin use in community studies: utility of serum thromboxane B_2_ Measurement. Clin Appl Thromb Hemost. (2014) 20:385–92. 10.1177/1076029613486537 23653145 PMC8088305

